# Lumbar Facet Joint Septic Arthritis Complicated by Spinal Epidural Abscess in Adolescents: Two Case Reports and a Literature Review

**DOI:** 10.7759/cureus.106397

**Published:** 2026-04-03

**Authors:** Zakariae Benyaich, Hajar Lahmidi, Kadiri A Naji, Karim Baayoud, Mohamed Lmejjati

**Affiliations:** 1 Neurosurgery, Mohammed VI University Hospital, Agadir, MAR; 2 Reins, Endocrinologie Gastro-Entérologie, Neurosciences et Ethique (REGNE) Laboratory, Faculty of Medicine and Pharmacy of Agadir, Ibn Zohr University, Agadir, MAR; 3 Dermatology, Mohammed VI University Hospital, Agadir, MAR; 4 Laboratory of Anatomy, Faculty of Medicine and Pharmacy of Agadir, Ibn Zohr University, Agadir, MAR

**Keywords:** adolescent, facet joint, mri, septic arthritis, spinal epidural abscess

## Abstract

Septic arthritis of the spinal facet joint (SAFJ) is a rare spinal infection predominantly affecting adults and remains exceptional in pediatric and adolescent populations. Spinal epidural abscess (SEA) may occur as a complication and can lead to severe neurological deficits if diagnosis and treatment are delayed.

We herein report two adolescent patients with lumbar SAFJ complicated by SEA. The first case involved a 14-year-old boy presenting with acute low back pain, fever, and rapid neurological deterioration culminating in cauda equina syndrome, requiring urgent surgical decompression and prolonged antibiotic therapy. The second case involved a 15-year-old girl presenting with acute low back pain and radiculopathy with a minor neurological deficit, successfully managed with conservative antibiotic treatment alone.

Clinicians should maintain a high index of suspicion for SAFJ in children and adolescents presenting with acute localized back pain and fever. Early MRI evaluation is crucial to detect facet joint infection and epidural extension before the onset of neurological deterioration. Prompt individualized management, guided primarily by neurological status, can result in excellent functional outcomes.

## Introduction

Septic arthritis of the spinal facet joint (SAFJ) is a rare but potentially severe spinal infection that may rapidly progress to spinal epidural abscess (SEA) [1,2]. Although predominantly reported in adults, pediatric and adolescent cases remain exceptional, with an estimated incidence of approximately 0.2 per 100,000 children per year, and pediatric-specific management guidelines are currently lacking [3,4].

The facet joint is a posterior synovial joint with a thin capsule that lies in close anatomical proximity to the posterior epidural space, which explains the propensity for epidural extension when infection occurs [1]. SEA is a neurosurgical emergency that can rapidly progress to paralysis and sepsis if not recognized and treated promptly [2,5].

Early symptoms are often nonspecific, typically presenting with localized back pain associated with fever or elevated inflammatory markers. This clinical ambiguity may lead to delayed diagnosis and treatment, increasing the risk of neurologic compromise [5,6].

Magnetic resonance imaging (MRI) is essential for the early detection of facet joint infection and epidural extension [7]. Prompt recognition and management guided by neurologic status are crucial to prevent irreversible deficits [1,3].

We report two adolescent cases of lumbar SAFJ complicated by SEA, illustrating two different therapeutic strategies: urgent surgical decompression versus conservative medical management. Through these cases and a review of the literature, we aim to highlight the importance of early recognition and individualized management in this rare pediatric condition.

## Case presentation

Case 1

A 14-year-old previously healthy boy presented with acute severe low back pain radiating to both lower limbs, associated with high-grade fever (39°C) and left paraspinal swelling. Within three days, he developed rapid neurological deterioration, including bilateral lower limb weakness and urinary retention. On admission to the Mohammed VI University Hospital of Agadir, neurological examination revealed signs consistent with cauda equina syndrome, including grade 3 paraparesis on the Medical Research Council (MRC) scale and saddle hypesthesia. Spinal examination showed focal left lumbar paravertebral tenderness to percussion.

Laboratory investigations revealed a markedly elevated C-reactive protein (CRP) level and leukocytosis (Table 1). HIV testing was negative.

**Table 1:** Evolution of inflammatory laboratory parameters during antibiotic treatment (Case 1).

Laboratory test	Reference range	Admission	Week 2	Week 4
White blood cell count (/mm³)	4,000-10,000	17,550	8,550	8,150
Neutrophils (%)	40-75	85	65	50
C-reactive protein (mg/L)	<6	303.7	20.2	<6

**Figure 1:**
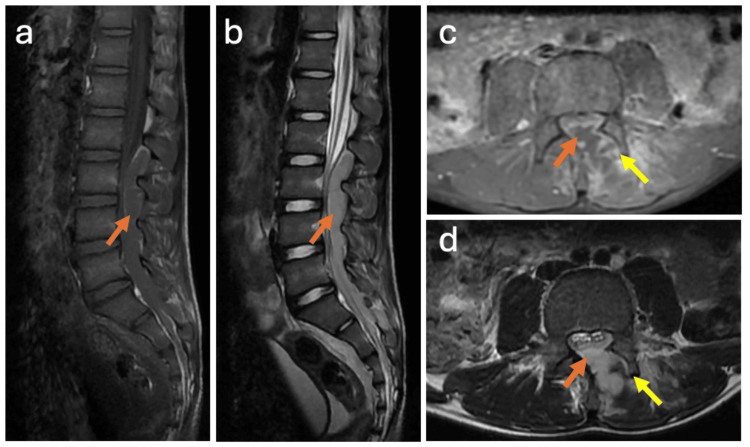
MRI of the lumbosacral spine demonstrating a posterior SEA arising from septic arthritis of the left L3-L4 facet joint (Case 1). Sagittal T1-weighted (a) and T2-weighted (b) images show a posterior epidural abscess extending from L2 to S2 (orange arrow), resulting in significant compression of the thecal sac and cauda equina nerve roots. Axial fat-suppressed contrast-enhanced T1-weighted images (c) demonstrate enhancement of the left L3-L4 facet joint (yellow arrow), the epidural collection (orange arrow), and the left paraspinal region. Axial T2-weighted images (d) show joint space widening and effusion of the left L3-L4 facet joint (yellow arrow) with epidural extension (orange arrow) and paraspinal muscle edema. SEA, spinal epidural abscess; MRI, magnetic resonance imaging

A prompt MRI study of the lumbosacral spine was performed and demonstrated left L3-L4 facet joint arthritis associated with a compressive posterior epidural abscess extending from L2 to S2 (Figure 1).

Emergency surgical intervention was undertaken. A left L3-L5 hemilaminectomy was performed, allowing evacuation of the epidural abscess and debridement of the infected facet joint. Immediate empiric intravenous antibiotic therapy with ceftriaxone, gentamicin, and metronidazole was initiated. Intraoperative cultures identified methicillin-sensitive Staphylococcus aureus (MSSA). The patient received four weeks of intravenous ceftriaxone followed by two weeks of oral levofloxacin.

Postoperatively, the patient experienced complete resolution of pain and motor deficits within the first week, with progressive recovery of saddle sensation and sphincter function. Inflammatory parameters were within normal range after four weeks of intravenous antibiotic therapy (Table 1). At 15-month follow-up, no recurrence or residual neurological deficit was observed.

Case 2

A previously healthy 15-year-old girl presented to Mohammed VI University Hospital of Agadir with acute low back pain and right L4-L5 radiculalgia, accompanied by fever (38.5°C). She reported an episode of streptococcal pharyngitis two weeks prior to presentation. Neurological examination revealed mild right foot dorsiflexion weakness (MRC grade 4) without sensory or sphincter disturbances. Spinal examination found lumbar spine stiffness and exacerbation of low back pain with extension.

Laboratory tests showed elevated CRP and leukocytosis (Table 2). Blood and urine cultures were negative.

**Table 2:** Evolution of inflammatory laboratory parameters during antibiotic treatment (Case 2).

Laboratory test	Reference range	Admission	Week 2	Week 4
White blood cell count (/mm³)	4,000-10,000	16,000	9,200	8,600
Neutrophils (%)	40-75	90	66	50.6
C-reactive protein (mg/L)	<6	170	11.3	<6

**Figure 2:**
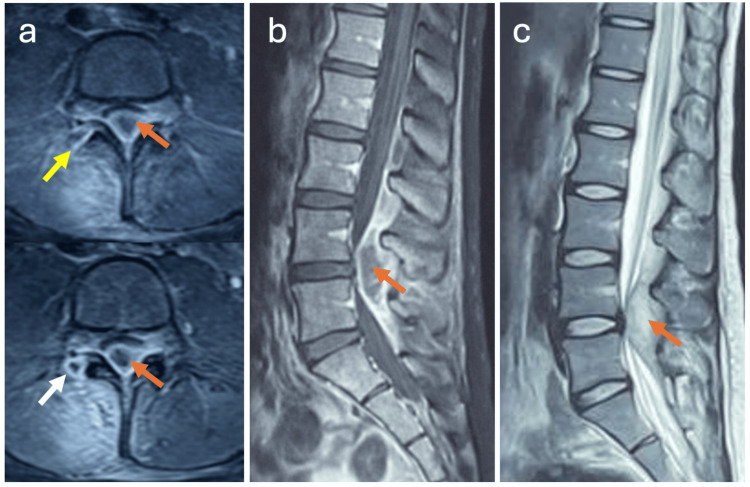
MRI of the lumbosacral spine showing a typical rim-enhancing epidural collection contiguous with the right L4-L5 SAFJ (Case 2). Axial fat-suppressed contrast-enhanced T1-weighted images (a) demonstrate enhancement of the right L4-L5 facet joint (yellow arrow) with a rim-enhancing lesion (white arrow) involving the articular process and an associated compressive epidural abscess. Sagittal contrast-enhanced T1-weighted image (b) and sagittal T2-weighted image (c) show a posterior epidural abscess (orange arrow) extending from L4 to L5, resulting in significant compression of the thecal sac and cauda equina nerve roots. MRI, magnetic resonance imaging

MRI of the lumbosacral spine demonstrated right L4-L5 facet joint arthritis with a posterior compressive epidural abscess (Figure 2). Given the absence of severe neurological deficits, the patient was managed conservatively with empiric intravenous antibiotics consisting of four weeks of ceftriaxone with five days of gentamicin, followed by two weeks of oral therapy with cefixime, along with analgesia and physiotherapy. Close clinical, biological, and radiological monitoring was maintained.

**Figure 3:**
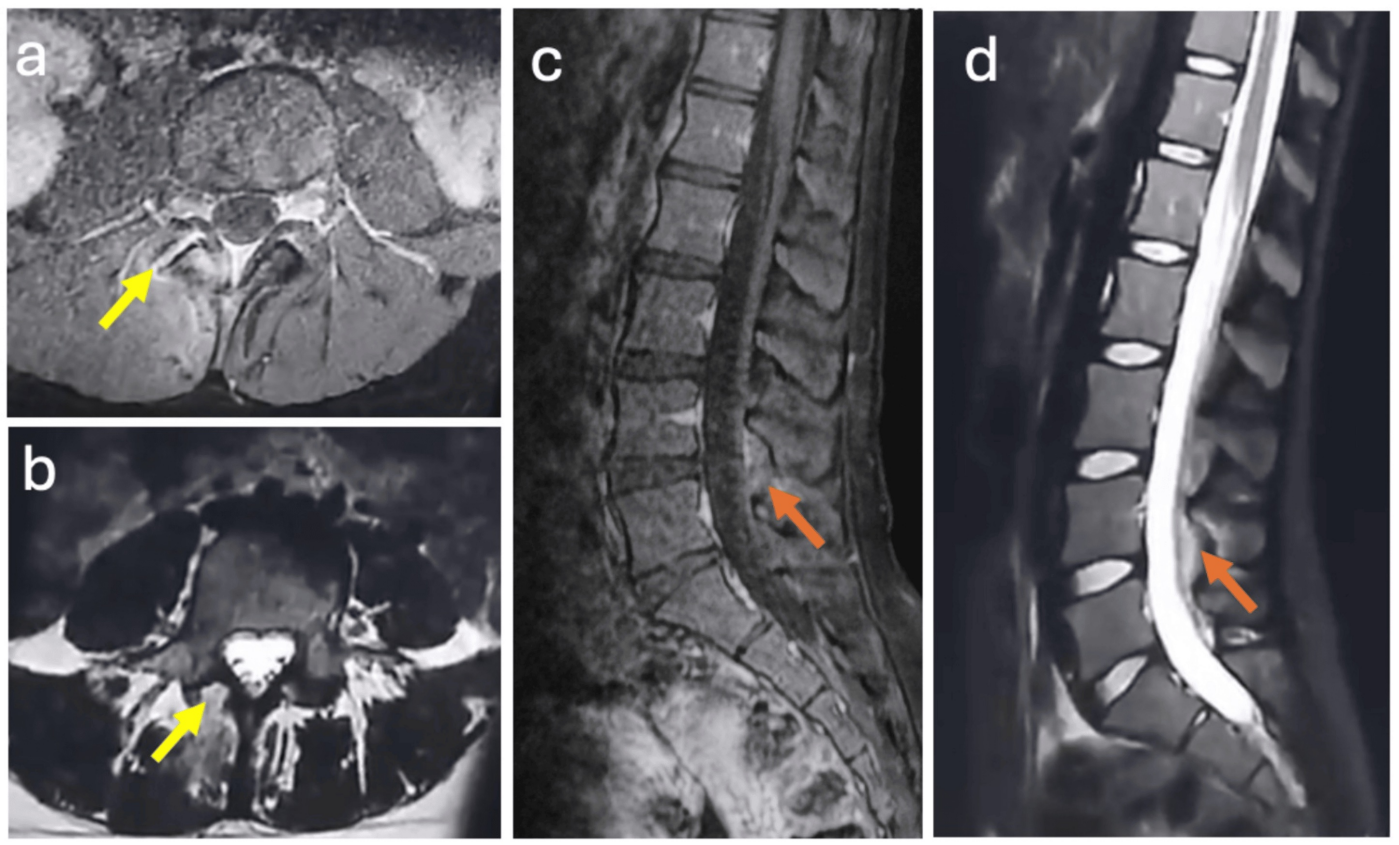
One-month follow-up MRI of the lumbosacral spine (Case 2). Axial fat-suppressed contrast-enhanced T1-weighted image (a) demonstrates persistent enhancement of the right L4-L5 facet joint (yellow arrow) without residual epidural collection. Axial T2-weighted image (b) shows hyperintense inflammatory signal changes involving the left inferior articular process of L4 (yellow arrow). Sagittal contrast-enhanced T1-weighted image (c) and sagittal T2-weighted image (d) demonstrate near-complete resolution of the epidural abscess, with residual inflammatory changes in the ligamentum flavum and interspinous soft tissues (orange arrow). These findings demonstrate radiological lag behind clinical improvement, with persistent MRI abnormalities despite complete clinical recovery. MRI, magnetic resonance imaging

The patient showed rapid clinical improvement, with complete pain relief, full neurological recovery, and normalization of CRP within two weeks (Table 2), supporting the adequacy of antibiotic therapy. Follow-up MRI at one month demonstrated a marked improvement with resolution of the epidural abscess (Figure 3). No recurrence was noted after six months of follow-up.

## Discussion

SAFJ is an uncommon spinal infection, accounting for approximately 1% of all cases of septic arthritis [4]. It predominantly affects adults over 50 years of age, particularly in the presence of comorbidities such as diabetes mellitus or immunosuppression [1,4]. In contrast, pediatric and adolescent cases are exceedingly rare and frequently underdiagnosed [3,5,6]. Although uncommon, reported cases span the entire pediatric age spectrum from infancy to late adolescence, with lumbar involvement representing the predominant localization [3].

Staphylococcus aureus remains the leading pathogen, responsible for up to 60% of infections, followed by streptococcal species [1,3]. As in our second case, blood culture-negative infections are not uncommon, highlighting the importance of obtaining tissue samples for microbiological analysis when surgical intervention is performed [3].

SAFJ is generally thought to result from hematogenous bacterial dissemination to the facet joint, which is a true synovial articulation containing a richly vascularized synovial membrane that facilitates bacterial seeding during episodes of transient bacteremia [1,8]. In our series, the second patient developed lumbar SAFJ shortly after a documented episode of streptococcal pharyngitis, supporting a possible bacteremic origin of the infection.

**Figure 4:**
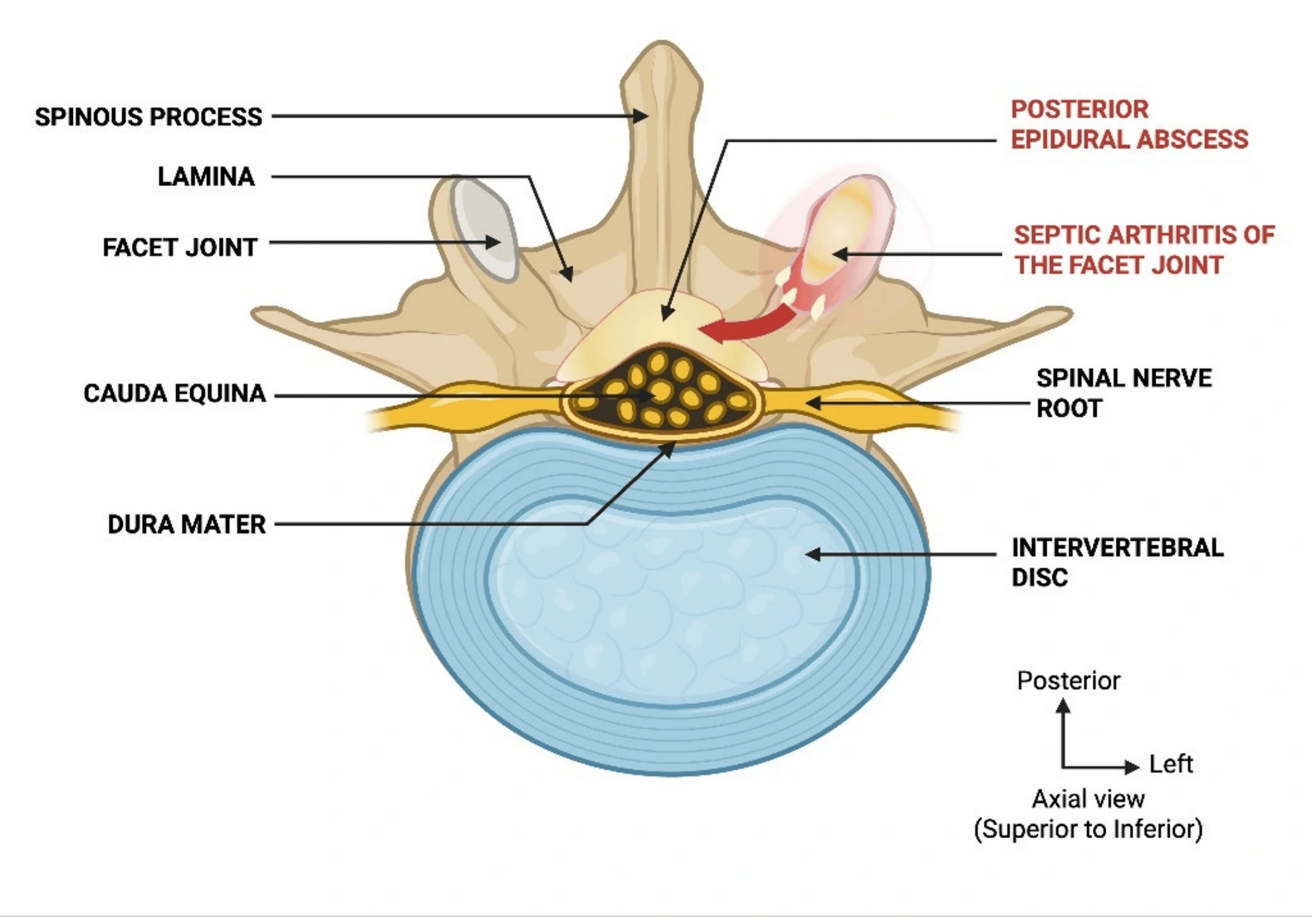
Anatomical diagram illustrating the pathway of infection spread from the lumbar facet joint to the epidural space. The diagram shows the anatomical relationship between the facet joint, the posterior epidural space, and the dural sac at the L5 level. Infection originating in the facet joint can extend anteriorly through the thin joint capsule into the epidural space (red arrow), leading to a SEA compressing the dural sac and the cauda equina nerve roots. SEA, spinal epidural abscess Created by authors using BioRender (BioRender.com Inc., Toronto, ON, Canada).

Once infection is established within the joint, it may extend to adjacent structures because of the anatomical characteristics of the facet articulation. The joint cavity is narrow and enclosed by a relatively thin capsule, allowing infection to spread beyond the joint. Anterior extension may occur through the ventral aspect of the capsule toward the posterior epidural space, leading to an epidural abscess (Figure 4), whereas posterior spread may involve the paravertebral muscles through disruption of the dorsal aspect of the capsule [9,10].

Clinical presentation is often nonspecific and may contribute to delayed diagnosis [10,11]. Localized acute back pain is the most consistent symptom, typically unilateral and aggravated by spinal extension or rotation, while fever is variably present [3,11]. Adolescents frequently present with focal low back pain and radiculopathy, whereas younger children may exhibit fever, limping, irritability, or refusal to ambulate [6,11-13]. Neurological deficits are uncommon in isolated SAFJ but are more likely in cases complicated by SEA, underscoring the importance of early recognition and prompt imaging [1,3,14,15].

MRI is the diagnostic gold standard and should be performed promptly whenever SAFJ is suspected [7,16,17]. Characteristic findings include facet joint effusion or widening, synovial enhancement, T2-weighted/Short Tau Inversion Recovery (STIR) hyperintensity of the articular processes, paraspinal edema or abscess, and posterior epidural abscess, often extending over multiple levels [7,17]. Importantly, normal findings on plain radiographs or computed tomography do not exclude the diagnosis and should not delay MRI evaluation [17].

Because available evidence in patients with SAFJ complicated by SEA is limited to isolated case reports and small series, standardized management guidelines have not yet been established [3,11,12]. Nevertheless, antibiotic therapy remains the cornerstone of treatment [1,3]. Empiric regimens should provide adequate coverage for Staphylococcus aureus and be subsequently tailored according to microbiological culture results when available [18]. Treatment duration typically ranges from four to six weeks, depending on clinical evolution and laboratory response [1,3,9]. The transition from intravenous to oral therapy is generally guided by clinical improvement, normalization of inflammatory markers, and radiological follow-up findings [3].

Surgical intervention is indicated in selected situations, including progressive or severe neurological deficits, failure of medical therapy, diagnostic uncertainty, or large epidural collections causing significant neural compression [2,18]. Notably, epidural extension alone does not systematically mandate surgical treatment in the absence of neurological compromise [3,18]. Both conservative and surgical strategies have been associated with favorable outcomes when carefully individualized (Table 3) [1,3]. The two cases reported herein illustrate this individualized therapeutic approach. The first patient required urgent surgical decompression because of severe neurological deficits (paraparesis, saddle anesthesia, and urinary retention), whereas the second patient, presenting with a mild isolated motor deficit, achieved complete recovery with antibiotic therapy alone despite the presence of a compressive epidural extension. Both cases showed no recurrence or neurological sequelae during follow-up, in contrast to findings reported in some pediatric SEA case reports and series [1,19].

**Table 3:** Comparative summary of pediatric cases of SAFJ with epidural abscess. mo, months; y, years old; M, male; F, female; LBP, low back pain; LL, lower limb; SAFJ, septic arthritis of the facet joint; SEA, spinal epidural abscess; MSSA, methicillin-sensitive Staphylococcus aureus; NA, not available; ATB, antibiotics

Authors	Age	Sex	Clinical signs	MRI findings	Organism	Treatment	Outcome
Le Hanneur et al. [12]	32 mo	M	Febrile limp, LBP	L5-S1 SAFJ, paraspinal inflammation	Kingella kingae	Surgery + ATB	Full recovery
Nakamura et al. [6]	25 mo	M	Fever only	Posterior SEA (T12-L3)	NA	ATB only	Full recovery
Heenan et al. [14]	10 y	M	LBP, fever, LL weakness, urinary symptoms	L4-L5 SAFJ + multilevel SEA	NA	ATB only	Full recovery
Maldonado-Morán et al. [9]	16 y	M	LBP, fever, headache, meningitis	L3 SAFJ + SEA	MSSA	ATB only	Full recovery
Vergine et al. [11]	13 y	F	LBP, radiculalgia, delayed fever	L4-L5 SAFJ + posterior SEA	MSSA	ATB only	Full recovery
Leng et al. [13]	10 mo	F	Fever, irritability, respiratory distress	Extensive cervical SEA	MSSA	Surgery + ATB	Full recovery
Prasad & De Vere [20]	14 y	F	Fever, abdominal pain, back pain	SEA T5-L3 + T7 SAFJ	NA	Surgery + ATB	Full recovery
Present case 1	14 y	M	LBP, fever, LL weakness, urinary retention	Lumbar SAFJ + large SEA	MSSA	Surgery + ATB	Full recovery
Present case 2	15 y	F	LBP, radiculalgia, fever	Lumbar SAFJ + posterior SEA	NA	ATB only	Full recovery

However, this report represents a single-center retrospective experience including only two cases, which limits generalizability. The rarity of pediatric SAFJ complicated by SEA precludes the development of standardized management guidelines. Large multicenter studies are required to better define optimal diagnostic and therapeutic strategies.

## Conclusions

SAFJ is a rare and often underdiagnosed condition, particularly in children and adolescents. The association of acute spinal pain and fever should prompt early MRI evaluation to assess facet joint involvement and epidural extension. Management must be individualized. Surgical treatment is reserved for patients with significant neurological compromise or failure of medical therapy, while conservative treatment may be effective in carefully selected, neurologically stable patients under close monitoring. With timely diagnosis and appropriate therapy, the prognosis of SAFJ with SEA is generally favorable in children, and full neurological recovery is often achievable.
